# Responsible Leadership and Affective Organizational Commitment: The Mediating Effect of Corporate Social Responsibility

**DOI:** 10.3389/fpsyg.2022.868057

**Published:** 2022-07-28

**Authors:** Rafael Alejandro Piñeros Espinosa

**Affiliations:** School of Management, Universidad del Rosario, Bogotá, Colombia

**Keywords:** corporate social responsibility, affective organizational commitment, responsible leadership, social identity theory, stakeholders

## Abstract

Organizations and their leaders are challenged to assume a responsible behavior given the increase of corporate scandals and the deterioration of employee commitment. However, relatively few studies have investigated the impact of responsible leadership (RL) on employee commitment and the effect of corporate social responsibility (CSR) in this relationship. Using the social identity theory this article examined the mediating effect of CSR practices in the relationship between RL and affective organizational commitment (AOC). Data collection was done through a paper survey completed by 309 full-time Colombian employees. Structural equation modeling was used to analyze the data. The results showed that CSR fully mediated the influence of RL on AOC. Thus, RL is an effective mechanism to develop CSR practices that in turn increase the levels of AOC of employees.

## Introduction

Corporate scandals and managerial misconduct have increased the need to reflect on the ethics and morality of corporate leaders (Voegtlin et al., 2012). Thus, there is a need to explain how those making the decisions in organizations impact corporate social responsibility (CSR) practices (Voegtlin, 2016). Because society has been losing trust in companies due to high levels of corruption, damage to stakeholders and the deterioration of natural resources it is said that we need a new conceptualization of the responsibilities of leaders (Patzer et al., 2018). Moreover, irresponsible leadership has been found to deteriorate the organizational commitment of employees (Boddy et al., 2010). These conditions suggest that society and employees demand a more responsible behavior on the part of companies and their leaders. To contribute to these issues, this research suggests that companies should promote a responsible leadership (RL) style in their managers as this favors the deployment of CSR practices that in turn increase the level of affective organizational commitment (AOC) of their employees.

On the other hand, the increase in environmental problems and the growing demands of stakeholders toward companies call for the redefinition of the responsibilities of their leaders (Maak and Pless, 2006). In the same way, it has been pointed out that companies must assume their social and environmental responsibilities (Scherer and Palazzo, 2011; Aguinis and Glavas, 2012). For these reasons, the need to develop responsible leaders has been indicated (Maak and Pless, 2009; Voegtlin, 2016).

Despite businesses efforts to find effective ways to incorporate CSR into their activities, research on the internal determinants of CSR, such as RL style, is limited (Aguinis and Glavas, 2012). Relatively few studies have investigated the relationship of RL with the psychological states of employees (Miska and Mendenhall, 2018). In particular, the association between RL and AOC, as well as the mechanisms that could explain this relationship, need further investigation (Haque and Caputi, 2017; Mousa, 2017). In this sense it has been argued that RL has a positive impact on AOC because employees will try to imitate responsible leaders’ behaviors as they give a sense of purpose and direction (Voegtlin et al., 2020). It has also been argued that leadership and CSR are personal and organizational factors considered key determinants of AOC (Meyer and Allen, 1991; Avolio et al., 2004; Rodrigo et al., 2019). To date no empirical studies have identified the effect of CSR in the relationship between RL and AOC. For these reasons, the objective of this study is to determine the effect of CSR practices in the relationship between RL and AOC in a group of Colombian employees.

## Literature Analysis and Hypothesis Development

### Literature Review

#### Responsible Leadership

The first approximation to the concept of (RL) was proposed by Lynham (1998) who explained that this style of leadership is oriented to achieve much more than economic results. It implies the adoption of a systemic thinking oriented to effectiveness, ethical behavior, and sustainability over time. Later, Doh and Stumpf (2005), explaining the need to connect leadership with CSR, defined it as a value-based leadership, characterized by ethical decision-making and quality relationships building with stakeholders.

The most cited definition in the literature is the one by Maak and Pless (2006). They indicate that RL is born from the recognition that companies must respond to various stakeholders. In their definition it is explained that RL is an ethical relational phenomenon with various interest groups, where the leader seeks to achieve a greater social good. The responsible leader not only influences his subordinates (employees) but builds long term relationships of trust and influence with various stakeholders (employees, clients, shareholders, suppliers, the government, and the community in general). Thus, they define the RL as: “the art of building and sustaining positive relationships with all relevant stakeholders, with the aim of coordinating actions and achieving common, sustainable and legitimate objectives” (p. 40).

Responsible leadership builds relationships through a process of social deliberation, involvement, and mobilization with stakeholders (Voegtlin et al., 2012) that improves the quality and legitimacy of decisions. In the context of a stakeholder society the purpose of RL is to contribute to sustainable development and the triple bottom line (Maak and Pless, 2006). Therefore, it has been indicated that the greatest challenge of RL is to get the organization to recognize and incorporate its social responsibility (SR) (Pless, 2007). Thus, the leader is responsible to various stakeholders, building relationships based on inclusion and facilitating dialogue between them to achieve a shared vision aimed at sustainable development. The involvement that this leadership style promotes with stakeholders generates the necessary knowledge to promote the innovations that allow the organization to survive and evolve (Doh and Quigley, 2014). Thus, Antunes and Franco (2016) explain that RL is a concept that emerges from the intersection that occurs between the studies of ethics, leadership, and CSR.

Conceptual discussions (see Waldman and Galvin, 2008; Waldman and Siegel, 2008) and discussions of empirical evidence (see Pless et al., 2012; Witt and Stahl, 2016), show that RL styles have different orientations. To enhance understanding and synthesize the RL phenomenon, Maak et al. (2016), explain two styles of RL from the theory of the upper echelons (Hambrick, 2007): instrumental and integrative. These two styles of leadership depend on the moral obligations that leaders perceive toward shareholders or stakeholders. The instrumental responsible leader perceives as a moral obligation the fiduciary duty assumed with the owners of the company. This instrumental approach conceives the role as the guardianship of the interests of the company’s owners. This role emerges as part of a psychological contract with the shareholders in which the leader considers them his employers. On the other hand, the integrative responsible leader, assumes that his moral obligation is with a broad set of stakeholders, and perceives a social contract between the company and society as valid, therefore, considers creating value for all stakeholders a responsibility. This does not mean that the integrative responsible leader does not care about economic performance; it is seen as the result of a successful and purposeful company. In this study we use the integrative approach of RL.

#### Corporate Social Responsibility

Corporate social responsibility has been addressed since the 1950s. It has been gaining relevance as organizations are pressured to contribute to the solution of environmental and social problems. According to Carroll (1999), the father of CSR is Bowen (1953). In his book published in 1953, “The Social Responsibilities of the Businessman,” he concluded that CSR implies the adoption of policies, decisions and actions that are desirable in terms of the objectives and values of society.

The interest in adopting CSR practices led the International Organization for Standardization (ISO) to develop the guide for incorporating SR practices (ISO 26000: Guidance on Social Responsibility) in organizations (ISO, 2010). In it, SR is defined as “the responsibility of an organization for the impacts that its decisions and activities cause on society and the environment, through an ethical and transparent behavior that contributes to sustainable development including the health and well-being of society …” (p. 4). This study adopted the definition of CSR proposed by Aguinis (2011), “Policies and actions in the organizational context that takes into account the expectations of stakeholders and performance based on the triple bottom line: economic, social and, environmental” (p. 855).

#### Affective Organizational Commitment

The study of the antecedents and consequences of employee commitment to the organization has been a topic of great interest and has been considered a connection or link between the individual and the organization (Mathieu and Zajac, 1990). AOC has particularly been investigated, as it relates to the emotional bond of employees with the organization. For example, work experiences and perceived organizational support have been found to positively influence AOC, which in turn positively impacts staff turnover rates (Rhoades et al., 2001). The meta-analysis by Meyer et al. (2002) concluded that of the three forms of commitment, AOC has had the strongest and most favorable correlations with behaviors such as performance, attendance, and organizational citizenship behaviors. Subsequent studies have found a relationship between organizational commitment and various measures of financial performance (Abdul Rashid et al., 2003).

Afterward, Mercurio (2015) concluded that the AOC is the historical and theoretical basis of the other types of commitment, and after carrying out a meta-analysis found that AOC positively affects the indicators of turnover, absenteeism, organizational citizenship behaviors, and stress. His findings lead him to conclude that AOC is the essence of organizational commitment. This is how AOC begins to be identified as one of the determinants of job performance (Sharma and Dhar, 2016; Wang et al., 2020) and as a mediator of the positive effect of human talent practices on the performance of business units (Raineri, 2017). More recently, it has been recognized as a mediator of the positive influence of supervisor feedback on innovative work behavior (Bak, 2020), and as a mediator of the positive effect of authentic leadership on individual creativity (Ribeiro et al., 2020).

### Hypotheses Development

#### Responsible Leadership and Affective Organizational Commitment

Different leadership styles have been related to employee commitment, this includes between others transformational leadership (Avolio et al., 2004; Keskes et al., 2018; Jiatong et al., 2022), servant leadership (Lapointe and Vandenberghe, 2018), spiritual leadership (Sapta et al., 2021), and authentic leadership (Ausar et al., 2016). This can be explained because several leadership behaviors like decentralization in decision-making, perceived organizational support, perception of importance for the organization (Meyer and Allen, 1991), and perceived organizational support (Rhoades et al., 2001; Meyer et al., 2002) have been identified as determinants of AOC. The RL carries out processes of employee involvement in decision-making, promoting participatory practices that allow the employee to feel important and committed (Voegtlin et al., 2012), also assumes management practices of organizational support to employees and human talent management (Doh et al., 2011), thus can be associated with the AOC by promoting participation and decentralization and increasing the perception of importance of employees for the organization.

In this research this relationship is explained through the social identity theory (SIT), that suggests that individuals tend to classify themselves in social categories that enable to define him or herself in the social environment, in this case the employees identify themselves with the RL role that serves as a referent (Ashforth and Mael, 1989). Particularly employees compare themselves with the dimensions of positive social value (Abrams and Hogg, 1990) that the RL demonstrate by having an ethical behavior and pursuing social and environmental goals.

On the other hand, RL behaviors are negatively related to intention to leave and staff turnover, and this relation is mediated by pride (Doh et al., 2011). RL has also been identified as a strong predictor of significant work in four dimensions: unity with others, expressing full potential, inspiration, and tension equilibrium (Lips-Wiersma et al., 2018). The effect of RL on organizational commitment have been found to be mediated by turnover intention (Haque and Caputi, 2017) and a climate of diversity and inclusion (Mousa, 2017). In this sense, a responsible employee will be linked to a responsible leader and the attributes of the group, which generates a feeling of identity and desire to remain in the company. For these reasons it is proposed that there may be a relationship between RL, and the AOC of employees as is stated in Hypothesis 1.

**Hypothesis 1:** Responsible leadership positively influences the affective organizational commitment of employees.

#### Responsible Leadership and Corporate Social Responsibility Practices

The relationships between value-based leadership styles such as transformational (Waldman et al., 2006), authentic (Iqbal et al., 2018; Kim et al., 2018b; Chaudhary, 2020), ethical (Nejati et al., 2020; Saha et al., 2020), and CSR practices have been studied. The findings indicate that, as these leadership approaches are based on strong ethical values, they can motivate employees by presenting CSR goals that align with their self-concept. Following SIT explanations of group membership, followers may identify with values that go beyond self-interest, like stakeholder needs and the needs of society (Waldman et al., 2006), and thus behave to achieve such goals.

According to the researchers of the RL field of study, there are several mechanisms that explain the RL-CSR relation, it has been explained that one of the central purposes of the RL is to ensure that companies incorporate CSR practices (Pless, 2007), and that the experience, values, and personality of the leader, shape their reasoning about the responsibility of the organization (Maak et al., 2016). Voegtlin et al. (2012) maintain that, RL promotes CSR practices through the construction of relationships with stakeholders, the promotion of an ethical culture based on deliberation practices and the process of raising awareness about the importance of CSR. Incorporating the concerns of stakeholders in decision-making allows the employees to understand the business purpose within the framework of CSR. Similarly, Stahl and Sully de Luque (2014) explain that the RL deploys actions to benefit and to avoid negative impacts on stakeholders. These behaviors of RL contribute to the development of CSR activities, the sense of identification and engagement of employees in responsible behavior with all stakeholders (Haque and Caputi, 2017).

On the relationship between leadership styles associated with RL and CSR, Godos-Díez et al. (2011) analyzed how the leader’s profile is related to the development of CSR practices, as well as the mediation of the perception of the role of ethics and SR; defining two leadership profiles: agency (characterized by selfishness and opportunistic behavior) and servant (characterized by cooperative behavior, which seeks to defend the well-being of various stakeholders) found that those managers with a servant profile were inclined to give more importance to the role and implementation of CSR. Some years later Castro González et al. (2017) identified a positive and significant relationship between RL and perceived CSR. Therefore, Hypothesis 2 is proposed.

**Hypothesis 2:** Responsible leadership positively influences corporate social responsibility practices.

#### Corporate Social Responsibility and Affective Organizational Commitment

Aguinis and Glavas (2017) recently studied how individuals proactively find meaning in their work, and how this is related to the way they experience CSR practices. In this sense, the relationship between CSR and AOC practices has been a topic of particular interest during the last decade, and several studies have indicated a positive effect of CSR on the organizational commitment of employees in various geographical locations such as North America (Peterson, 2004; Glavas and Kelley, 2014; Vlachos et al., 2014), Pakistan (Ali et al., 2010; Asrar-ul-Haq et al., 2017), Europe (Ditlev-Simonsen, 2015; Mory et al., 2016a,b), Africa (Mensah et al., 2017; Bouraoui et al., 2018), India (Gupta, 2017), and South Korea (Kim et al., 2018a). As part of the GLOBE project, Mueller et al. (2012), analyzing information collected in 17 countries and with 1084 employees, found a positive relationship between perceived CSR and AOC; their analyzes show that this relationship is strengthened in cultures where there is greater institutional collectivism, human orientation and that it weakens when there are high levels of power distance.

Another stream of research that emphasizes the multidimensionality of CSR has identified the relationship between CSR components and AOC. Internal and external CSR have showed positive effects on organizational commitment (Brammer et al., 2007) as well as CSR to social and non-social stakeholders, to employees and customers (Turker, 2009a). Additionally, AOC has been found to be influenced by CSR related to education and training, human rights, health, safety at work, work life balance and diversity at work (Al-bdour et al., 2010), by CSR with the community, consumers, and employees (Farooq et al., 2014), by CSR with employees, customers, and the government (Hofman and Newman, 2014) and CSR oriented to health, safety, education, and training (Thang and Fassin, 2017).

The relationship between CSR and AOC practices, is explained using the SIT that suggests that employees associate aspects of their self-concept with behaviors and attitudes of certain social groups (Turner and Oakes, 1986). In this case the employees with values oriented toward SR will feel more emotionally committed to organizations that carry out CSR practices, generating a bonding process. Therefore, Hypothesis 3 is proposed.

**Hypothesis 3:** Corporate social responsibility practices positively influences the affective organizational commitment of employees.

#### Corporate Social Responsibility as a Mediator of the Relationship Between Responsible Leadership and Affective Organizational Commitment

In this research, it is proposed that a variation in the RL can cause a variation in the perceived CSR, which in turn could generate a higher level of AOC. This conjecture is argued as follows: the RL creates value for a range of stakeholders in business and in society (Pless et al., 2012), leads the company with an emphasis on the triple bottom line and justifies their decisions under a logic of what is appropriate (Maak et al., 2016). The RL influences the CSR character of the organization, making employees aware of the possible social and environmental consequences of corporate actions, by emphasizing and demonstrating with their actions the importance of involvement with different stakeholders (Voegtlin et al., 2012). These behaviors lead to higher levels of AOC in the employees as their social identity is enhanced when the organization to which the employee belongs is distinctive and more positive than other organizations (Allen et al., 2017).

Thus, RL orients decisions and behavior toward responsibility and coordinates actions to achieve a shared vision of CSR (Maak, 2007). Evidence of this relationship is found in the research carried out by Castro González et al. (2017) that signaled a positive and significant relationship between RL and perceived CSR. In turn, the adoption of CSR practices has been shown to be positively related to organizational commitment (Brammer et al., 2007; Turker, 2009b; Mueller et al., 2012; Hofman and Newman, 2014). The empirical findings of related research explain that, according to the SIT, responsible employees identify themselves with a company that implements CSR practices and has responsible leaders. Therefore, Hypothesis 4 is proposed.

**Hypothesis 4:** CSR is a mediator of the relationship between responsible leadership (RL) and affective organizational commitment (AOC).

**FIGURE 1:**
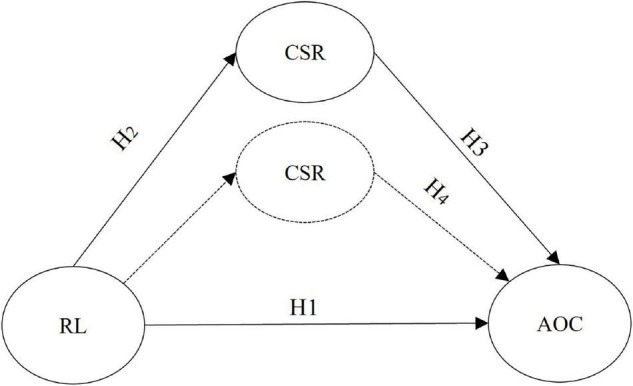
Hypothesized model proposing the direct and mediational relationships. RL, responsible leadership; CSR, corporate social responsibility; AOC, affective organizational commitment.

From the previous discussions, a hypothesized model for this study is depicted in Figure 1.

## Materials and Methods

### Methodological Approach

This research aimed to determine if the RL style has a direct effect on organizational affective commitment (AOC) or if this relationship is mediated by CSR practices. The cause-and-effect explanation makes it part of the functionalist-positivistic research paradigm described by Burrell and Morgan (1979). This study is descriptive, correlational, and explicative in nature. From the perceptions of employees recollected in one point of time, the behaviors of RL, the CSR practices, and the level of AOC are estimated.

In this study, RL influences CSR which in turn influences AOC; it has been suggested that to test this type of causal structures the technique of structural equation modeling (SEM) is adequate (Hair et al., 2009). SEM has been signaled as a robust technique due to its ability to control measurement errors, the possibility of handling different dependent variables and testing models with different assumptions of causality (Ramlall, 2017). It has been considered appropriate to verify if the hypothesized theoretical model is adequate for the sample data (Thakkar, 2020).

### Data Collection Methods

The subject type sampling method was used in this study. As inclusion criteria, the participants were Colombian employees, working in the same organization with the same leader for at least the last 12 months, and with a full-time contract. The employees belonged to organizations of different economic sectors and sizes, occupied jobs in different hierarchical levels and different professional backgrounds, allowing variability regarding the type of leader and organization evaluated. This diversity minimizes the common method bias (CMB) as it’s explores different organizational contexts (Podsakoff et al., 2003).

The scales originally developed in English were back translated from Spanish following Brislin (1970) procedure. To minimize CMB, two blank lines were inserted between each scale, a specific instruction was given before the presentation of each set of questions and different number of Likert scale-points were used for each measurement instrument. These procedures allow to psychologically separate the measurement of the independent and dependent variables (Podsakoff et al., 2003). Complementarily, to enhance the motivation to answer the questions, a common and precise language was used, defining less-familiar concepts as could happen with the term: “stakeholders.” Printed questionnaires had spaces between items to eliminate the proximity effect. The data was collected in different parts of the country and analyzed in the SPSS statistical software. To run the SEM procedures the AMOS package was used. Data recollection was done during the second semester of 2019, and during January and February of 2020; 640 questionnaires were answered, of which 309 complied with the inclusion criteria and were considered valid. Finally, the participants were told that their participation was anonymous voluntary, and that they could retire from the study at any time.

In relation to the demographic profile, 55.3% of the survey respondents were women and 44.7% were men. The age of the respondents ranged from 18 to 40 or above, with a 42.7% for 18–29 years and with 44.7% for 30–39 years. Among all respondents, 73.8% completed technical or professional education and 26.2% postgraduate degrees. Lastly, 50.6% of respondents had worked with the same superior for 12–28 months and 49.5% for more than 28 months. The detailed data is presented in Table 1.

**TABLE 1:** Sociodemographic characteristics of participants.

Feature	Frequency	Percentage
**Gender**		
Female	171	55.3
Male	138	44.7
Total	309	100.0
**Age group (years)**		
Between 18 and 29	132	42.7
Between 30 and 39	138	44.7
40 or more	39	12.6
Total	309	100.0
**Education level**		
Technical and professional	228	73.8
Postgraduated	81	26.2
Total	309	100.0
**Time working with the immediate manager (months)**		
12 and 28	156	50.5
More than 28	153	49.5
Total	309	100.0

### Measures

In the case of RL the unidimensional scale developed by Voegtlin (2011), was selected as it has shown appropriate levels of reliability (Voegtlin, 2011; Castro González et al., 2017; Han et al., 2019; Zhao and Zhou, 2019). This scale is comprised of five items with a 5-point Likert scale: 1. Never, 2. Rarely, 3. Every once in a while, 4. Sometimes, 5. Almost always.

To operationalize and measure CSR practices the scale developed by El Akremi et al. (2018) was used. The scale evaluates the CSR perception of employees using 35 items in relation with the following stakeholders: employees, customers, the environment, shareholders, suppliers, and the community. This instrument has a 6-point Likert scale: 1. Strongly disagree, 2. Disagree, 3. Somewhat disagree, 4. Somewhat agree, 5. Agree, 6. Strongly agree.

To measure AOC the scale developed by Mory et al. (2016b) was selected because it is an improved version of the one built by Meyer and Allen (1991). Its unidimensional and has eight items evaluated through a 7-point Likert Scale: 1. Strongly disagree, 2. Disagree, 3. Somewhat disagree, 4. Neither agree nor disagree, 5. Somewhat agree, 6. Agree, 7. Strongly agree.

### Methods of Analysis

The variables RL, CSR, and AOC, were analyzed using descriptive and correlational statistics. To test the proposed hypothesis, the two steps procedure suggested by Byrne (2010) for the SEM technique were carried out: assessing the measurement model and developing the structural model. The first step is performed to examine the validity and reliability of each of the measurement instruments and in this study was developed through a confirmatory factor analysis (CFA). The second step is to test the hypothesized structural model to see if it fits with the data from the sample, this was done using the AMOS module of SPSS. To assess data normality, skewness and kurtosis indicators were calculated. As the data showed a multivariate non-normal distribution, the “Bollen-Stine Bootstrap” (Bollen and Stine, 1992) procedure was carried out to determine if the model was acceptable and SEM fit indexes were estimated using the procedure for non-normal data distributions proposed by Walker and Smith (2017). To determine the significance of the indirect effect of RL through CSR on AOC, a Bootstrap with 5000 iterations and a confidence interval of 95% was executed following Byrne (2010) procedure.

This study considered three control variables: organization size as larger organizations have been found to develop more CSR practices than small ones (McWilliams and Siegel, 2001), sex as previous studies have identified women to score higher on AOC than men (Brammer et al., 2007) and geographical scope, as it is expected that multinational organizations are more willing to perform CSR practices than local ones. The Harman’s one-factor test was calculated to assess if the CMB affected the proposed model.

## Results

To determine if the RL directly affects the AOC or if this relationship is mediated by CSR, in this section the results are detailed in the following order: first the descriptive and correlation statistics of the principal variables are showed, then the results of the SEM are presented. The results of the effects of the control variables and of the single factor test for the hypothesized model are subsequently described. Finally, the results of the mediation test are presented.

### Descriptive Statistics and Correlation Test

To identify associations between variables Pearson’s correlation coefficients were calculated. Table 2 allows us to identify that in the study the highest correlation between the main variables is between CSR and AOC with a coefficient of (0.54; *p* < 0.01), followed by the coefficient (0.31; *p* < 0.01) between RL and CSR. On the other hand, the weakest coefficient among the three (0.25; *p* < 0.1) is between RL and AOC.

**TABLE 2:** Pearson’s correlation coefficients.

	RL	CSR	CSR community	CSR environment	CSR employees	CSR suppliers	CSR clients	CSR shareholders	AOC
RL	1								
CSR	0.31**	1							
CSR community	0.03	0.55**	1						
CSR environment	0.29**	0.83**	0.50**	1					
CSR employees	0.34**	0.80**	0.22**	0.62**	1				
CSR suppliers	0.30**	0.80**	0.20**	0.53**	0.63**	1			
CSR clients	0.22**	0.77**	0.14**	0.50**	0.65**	0.69**	1		
CSR shareholders	0.22**	0.71**	0.08	0.43**	0.56**	0.60**	0.64**	1	
AOC	0.25**	0.54**	0.16**	0.39**	0.51**	0.49**	0.44**	0.47**	1

*n = 309.*

**The correlation is significant at the 0.01 level (bilateral).

#### Structural Equation Modeling

As is detailed in Appendix Table 1, the (β2) values indicated that none of the items has significant kurtosis (<7), however, the critical ratio exceeds for various items the critical value *z* (±1.96) signaling a multivariate non-normal data distribution (Byrne, 2010). Since the multivariate non-normal distribution can alter the standard error of the coefficients between the latent variables in SEM (Andreassen et al., 2006), and underestimate the Tucker–Lewis index (TLI) and comparative fit index (CFI) fit indices when the maximum likelihood estimation method is used (Byrne, 2010), the procedure known as “Bollen-Stine Bootstrap” (Bollen and Stine, 1992) was carried out to determine if the model can be accepted. Subsequently, the two procedures proposed by Byrne (2010) were carried out; the first step was the evaluation of the measurement model and the second the development of the structural model. During these procedures, the SEM adjusted indices for non-normal data were calculated according to the indications of Walker and Smith (2017). Accordingly, initially the results of the CFA are detailed for the measurement model and afterward the results obtained for the structural equation model (SEM) is presented.

#### First Step: Measurement Model

In this study, the RL was measured using the scale developed by Voegtlin (2011) with five items to consider the employees’ perception of RL. The perceptions of CSR practices by the employees was measured with 35 items of the instrument developed by El Akremi et al. (2018) with a Cronbach’s alpha for this study of (α = 0.95). The instrument is made up of six subscales: CSR with the community (α = 0.91), CSR with the environment (α = 0.90), CSR with employees (α = 0.89), CSR with suppliers (α = 0.89), CSR with customers (α = 0.87), and CSR with shareholders (α = 0.91). The AOC was measured with the one-dimensional 8-item scale developed by Mory et al. (2016b). Since the data indicated a non-normal multivariate distribution, the bootstrap procedure was executed (Bollen and Stine, 1992) and a *p*-value = 0.025 less than 0.05 was obtained, which indicated that the model was not consistent with the data. When reviewing the factor loadings of each of the first order dimensions in the second order dimension CSR, it was identified that the dimension “CSR with the Community” presented a standardized factor load of 0.21, so following the indications of Hair et al. (2009) was eliminated from the measurement model. With the new measurement model without the “CSR with the Community” dimension, the bootstrap procedure was executed (Bollen and Stine, 1992) and a value of *p* = 0.106 greater than 0.05 was obtained, which indicates that this model was consistent with the data. To calculate the fit indices, the results of χ^2^ = 10461.674 and df = 820 of the independence model, those of χ^2^ = 1040.725 and df = 708 of the base model, the sample size *n* = 309 and the value *p* = 0.106 were used to run the procedure established by Walker and Smith (2017). The model fit indicators are presented in Table 3.

**TABLE 3:** Goodness of fit indices of the measurement model.

Unadjusted and adjusted Chi-square statistics and scaling factor					
	**Chi-square statistic**	**Bollen–Stine Adjusted Chi-square equivalent statistic**	**Bollen–Stine Scaling factor**		

	1040.725	755.317	1.378		

**Unadjusted and adjusted goodness-of-fit indices**					

**CFI**	**Adjusted CFI**	**TLI**	**Adjusted TLI**	**IFI**	**Adjusted IFI**

0.965	0.995	0.960	0.994	0.966	0.995

**Unadjusted and adjusted residual fit indices**					

	**RMSEA**	**Adjusted RMSEA**			

	0.039	0.015			

*SEM, structural equation modeling; RMSEA, root mean square error of approximation; CFI, comparative fit index; TLI, Tucker–Lewis index; IFI, incremental fit index.*

**FIGURE 2:**
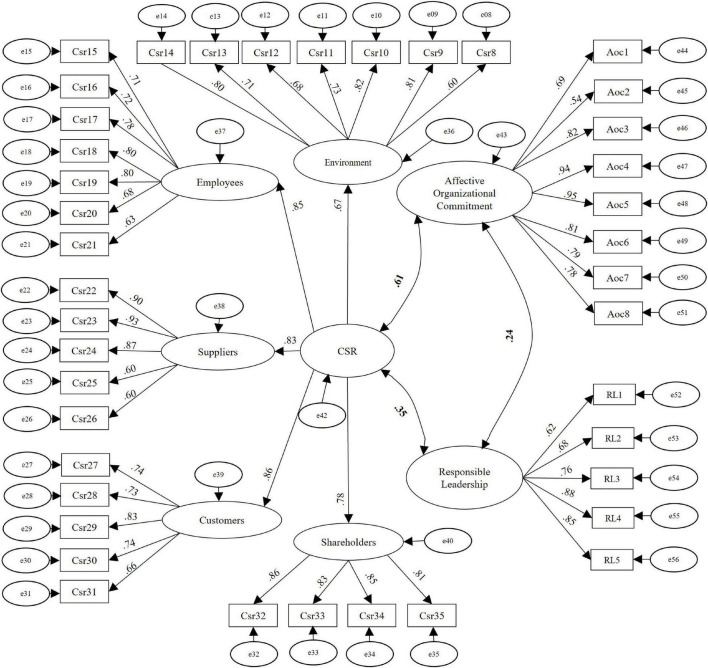
Second-order confirmatory factor analysis of the measurement model. This figure represents the CFA of the measurement model, the values in bold correspond to correlations and the others to standardized factor loadings of the observed and latent variables, they are all significant (*p* < 0.001). *n* = 309.

Indices greater than 0.95 were obtained in the adjusted indices of CFI, TLI, and incremental fit index (IFI), and a value less than 0.08 for the adjusted root mean square error of approximation (RMSEA), which indicates that the measurement model adjusted to the data adequately. The results of the CFA are presented in Figure 2 indicating levels of significance and adequate standardized factor loadings (Hair et al., 2009).

Thus, the CFA results for the measurement model present appropriate fit indicators and factor loadings.

#### Second Step: Structural Models

**FIGURE 3:**
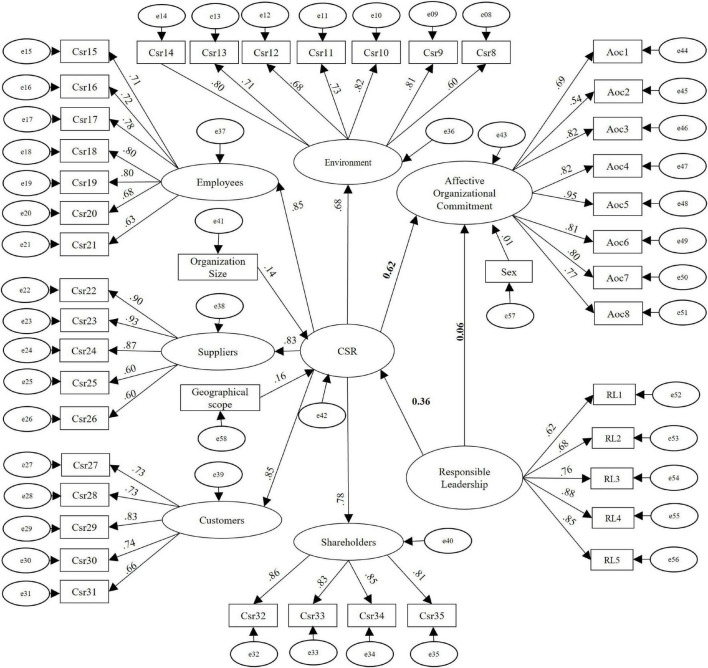
Structural equation model of the mediation of CSR between RL and AOC. The values in bold correspond to standardized coefficients (β) and the others to standardized factor loadings of the observed and latent variables. *n* = 309.

In this study, a structural model was developed to test the mediating effect of CSR on the effect between RL and AOC. The standardized regression coefficients (β) are presented in bold in Figure 3.

Since the data showed a non-normal distribution, the bootstrap procedure was executed (Bollen and Stine, 1992), obtaining a value *p* = 0.066 greater than 0.05, which indicates that the model is consistent with the data. To calculate the fit indices, the results of χ^2^ = 10681.241 and df = 946 of the independence model, those of the base model χ^2^ 1229.038 and df = 830, the sample size *n* = 309 and the value *p* = 0.066 were used to run the procedure established by Walker and Smith (2017). The model fit indicators are presented in Table 4.

**TABLE 4:** Goodness of fit indices of the hypothetized model.

	Hypothesized model*
**Absolute fit**	
*x* ^2^	892.196
**Comparative fit**	
CFI	0.994
TLI	0.993
IFI	0.994
**Others**	
RMSEA	0.016

*The values presented in the hypothetized model, correspond to adjusted indices for the non-normal multivariate dada following the procedure suggested by Walker and Smith (2017).

Indexes greater than 0.95 were obtained in the adjusted indices of CFI, TLI, and IFI, and an index less than 0.08 for the adjusted RMSEA, which indicates that the CSR mediation model adjusted to the data adequately.

The hypothesis was tested using the estimated parameters of the structural model. As presented in Table 5, the direct effect of RL on AOC was not significant (β = 0.06; *p* > 0.005), rejecting Hypothesis 1, while the indirect effect of RL on AOC, through CSR, was (β = 0.22; *p* < 0.001) which confirmed Hypothesis 4. The significance of the indirect effect was calculated through the Bootstrap procedure with 5000 samples and a 95% corrected bias confidence interval in the AMOS software. The effect of RL on CSR was positive (β = 0.36; *p* < 0.001), confirming Hypothesis 2. A positive influence of CSR on AOC is also observed (β = 0.62; *p* < 0.001), confirming Hypothesis 3.

**TABLE 5:** Results for the hypotheses to be tested in the CSR mediation model.

				Bound values		
Hypothesis		Direct effect	Indirect effect	Lower	Upper	Results of the analysis
H1	RL → AOC	β = 0.06*^ns^*		−0.06	0.18	Not accepted
H2	RL → CSR	β = 0.36***		0.22	0.49	Accepted
H3	CSR → AOC	β = 0.62***		0.49	0.73	Accepted
H4	RL → CSR → AOC		β = 0.22***	0.13	0.33	Accepted

The β values are standardized coefficients; ^ns^not significant; ***p < 0.001.

As control variables for this research, the size, geographical scope of the organization (local or multinational) and the sex of the employees surveyed were considered. As can be seen in Figure 3, the size of the organization has a standardized effect on CSR practices of (β = 0.14; *p* < 0.05); the geographic scope of operation a standardized effect on CSR practices of (β = 0.16; *p* < 0.01); and finally, the sex of the participants has a standardized effect of (β = 0.16; ns). To determine the existence of the common method bias, the Harman single factor test was carried out, which assesses the degree to which a latent common factor accounts for all the manifest variables. The test was carried out using an exploratory factor analysis with an unrotated factor solution (Podsakoff et al., 2003). The total variance explained by a single factor was 36.8%, less than 50%, which indicates that the common method bias was not a risk in this study.

## Discussion

The purpose of this study was to determine the effect of CSR practices in the relationship between RL and AOC in a group of Colombian employees, the results showed that RL influences the level of AOC through the development of CSR practices. This demonstrate that RL can be a relevant determinant of value generation for stakeholders and the environment. Also, that it can be a leadership style that emotionally connects the employee to the organization, through the development of CSR practices.

The claim of contributing to the challenge of sustainability from management science (Ghoshal, 2005) implies the comprehension of the leadership needed to manage environmental and social issues in organizations. Ethical scandals of several managers, environmental movements, and social expectations, have seriously questioned the vision and behavior of business leaders (Muff et al., 2020). For these reasons, this research expands the existing knowledge in the field of RL, describes it as aware of the economic, environmental, and social impact of organizations, and points out the implications of promoting spaces for collective construction with stakeholders. It is emphasized in this research that the RL not only focuses on the relationship of influence with its employees, but also builds long-term and trusting relationships with multiple stakeholders (Maak and Pless, 2006). This conception allows to understand the role of business leaders as global citizens who seek the common good (Maak and Pless, 2009) and the dimension of responsibility in managerial practice (Voegtlin, 2016). This is how the RL is conceived responsible toward a wide set of interest groups (Maak et al., 2016) being the one that maximizes value for the different interest groups, internalizes the negative impacts, is long-term oriented and is regenerative rather than degenerative (WBCSD, 2020).

This research expands the knowledge on the outcomes of RL. It has been pointed out that the RL deploys CSR practices (Voegtlin et al., 2012) and this study presents empirical evidence of the positive and significant effect of the RL style on the development of CSR practices. This result indicates that those leaders who consider the concerns of stakeholders in their decision-making process and who seek to generate value in the triple bottom line contribute to the deployment of various CSR activities. According to SIT the followers will be proud of their responsible leaders, sharing a social category membership with the organization, and acting accordingly. It provides additional evidence to the positive influence of RL on CSR found by Castro González et al. (2017) in Spain and to the investigations that account for the relationship between leadership styles and the development of CSR practices (Godos-Díez et al., 2011; Groves and LaRocca, 2011; Du et al., 2013; Saha et al., 2020). It provides complementary evidence of the positive relationship between leadership and organizational commitment in the Colombian context (Bohorquez, 2016; Mañas-Rodríguez et al., 2020).

In this study, the understanding of the effects of CSR is broadened, by confirming hypothesis number three that indicates the positive and significant effect of CSR practices on the AOC level of employees in Colombia. This is justified as SIT suggest that individuals have a desire for positive self-evaluation, in this case being part of an organization that behaves more responsibly than others can enhance their social identity (Abrams and Hogg, 1990). This result is in line with the findings on the positive influence of CSR on AOC in other geographical areas such as North America (Peterson, 2004; Glavas and Kelley, 2014; Vlachos et al., 2014), Pakistan (Ali et al., 2010; Asrar-ul-Haq et al., 2017), Northern Europe (Ditlev-Simonsen, 2015; Mory et al., 2016a,b), Africa (Gupta, 2017; Mensah et al., 2017; Bouraoui et al., 2018), South Korea (Kim et al., 2018a), and in the countries that have been part of the GLOBE project (Mueller et al., 2012). According to the literature review, this increase in AOC levels is due to higher levels of identification and reciprocity of the employee with the organization that is concerned with generating social, environmental, and not only economic value. This finding is in line with the positive and significant relation between internal CSR and AOC found the Colombian context (Ávila-Tamayo and Bayona, 2022). However, the use of the multidimensional CSR scale, developed by El Akremi et al. (2018) allowed to identify that the CSR dimension with the community was not relevant in the Colombian context; its slight manifestation may be due to organizations prioritizing other CSR dimensions. This study contributes to the body of knowledge of CSR by identifying RL as an antecedent and AOC as an outcome.

Furthermore, this research contributes to the understanding of the mechanism of influence of RL on AOC. The result of the parameter that evaluates Hypothesis 1 indicates that the direct influence of RL on AOC is almost null and not significant; while the test parameter for Hypothesis 4 indicates that the indirect effect of RL on AOC through CSR practices is positive and significant. The result of Hypothesis 1 indicates that the variations in AOC levels are not directly explained by the adoption of the RL style. According to the result of Hypothesis 4, employees will increase their AOC level when the RL achieves the effective deployment of CSR practices. This finding can be explained using the SIT (Turner and Oakes, 1986), according to which people are attracted to groups whose behaviors are framed by what they consider valuable. In this way, organizations that incorporate or develop managers with RL styles will be able to deploy CSR practices, which will increase the AOC level of employees. This finding is consistent with the results of the research carried out by Castro González et al. (2017) who identified that CSR mediates the influence of RL on the creativity of a group of vendors, those of Mousa (2017) who found that climate diversity and inclusion mediates the relationship of influence between RL on organizational commitment, those of Haque and Caputi (2017) which indicate that employee turnover intention partially mediate the effect of RL on the AOC level and those of Voegtlin et al. (2020) that found a positive relationship between RL and AOC. The result is also associated with the mediating role of CSR in the relationship between transformational leadership and AOC reported by Allen et al. (2017). In general, it is observed that the effects of the RL style on the psychological and behavioral states of employees occur indirectly. Likewise, it was observed that gender did not affect the AOC levels, while the size of the organization and the geographical scope of operation affected the level of CSR practices in a slightly but significant way.

### Implications of the Study

In terms of theoretical implications, this study provides an explanation of how CSR mediates the relationship between RL and AOC using the SIT framework. Followers feel identified with responsible leaders that act according to higher moral standards, and with organizations that are responsive to societal expectations. These higher levels of social identification with RL and organizations increase the level of AOC. This study explains how CSR practices mediate the relationship as employees are the first ones to experience the effects of RL in the operations of the organization and their impact in the wellbeing of society and the planet.

The results of this research have practical implications for managers who face the challenge of getting their organizations to regain the trust lost due to negative environmental impacts and increased inequity (Edelman, 2020). The findings indicate that managers who adopt and promote the RL will favor the development of CSR practices in their organizations, building sustainable and trusting relationships with different stakeholders and coordinating their action to achieve common goals, sustainability, and social legitimacy (Maak and Pless, 2006). Besides, organizations should recognize that developing RL behaviors can strengthen the capacity of managers to motivate and have an engaged workforce, for example in the Colombian context adopting a transformational leadership style – ethical and value-based leadership approach – can help managers increase the level of commitment of their workers (Bohorquez, 2016; Mañas-Rodríguez et al., 2020). Additionally, as organizations are pressured to contribute to sustainable development, HR departments should include responsible competencies as criteria for selecting leaders; and develop leadership training programs that emphasize the development of RL skills.

On the other hand, since the results of this research indicate the strong influence of CSR practices on the AOC of employees, it is desirable to periodically socialize the projects that the company carries out to meet the needs of the different stakeholders. Previous research has found that workers with high levels of AOC improve their job performance (Sharma and Dhar, 2016; Wang et al., 2020) and achieve higher levels of creativity (Ribeiro et al., 2020). The results also indicate that it is not enough for managers to recognize and show their willingness to incorporate CSR, it is necessary to effectively deploy CSR projects in such a way that employees perceive their development, which in turn enhance their self-identity and levels of AOC. For the Colombian context it has been found that is desirable to develop internal CSR practices to develop higher levels of AOC (Ávila-Tamayo and Bayona, 2022).

These findings suggest that managers that wish to develop CSR practices can adopt a RL style, and that this leadership style can increase AOC when CSR practices with employees, suppliers, customers, and the environment are developed. This work provides a new conceptual model which considers the mediation role of CSR on the relationship between RL and AOC and offers an empirical validation with a sample of employees in a developing country.

### Limitations and Future Research

This study has limitations related to data collection, sample size and scope. Data collection was carried out through a self-report questionnaire answered by employees, which can lead to social desirability bias; this means that the participants could tend to present a favorable image of themselves and their organizations. In future studies, this bias can be mitigated by using various sources for data collection, for example, surveying not only employees but also managers. Since it is a cross-sectional study, it does not allow the analysis of data evolution. Future studies could use a longitudinal approach that provides more complete explanations on the causality between variables under study.

This investigation used a subject type – feasible sample so a larger sample is desirable in further research to generalize. For example, future studies could use representative samples from various sectors or countries to evaluate the effect of context variables such as culture, sector dynamics or government regulations.

The hypothetical model could be refined to give more rigor to the study, for example, it is necessary to evaluate the probable mediating role of human resources practices such as: job design, organizational climate, or work life balance. Research on the effects of RL on other outcomes such as civic or environmental behaviors is needed. On the other hand, research on internal determinants of CSR, such as board composition or strategic choices is still required. Finally, future research from a qualitative approach could investigate the reasons why certain dimensions of CSR are presented with more intensity than others and how they are developed.

## Data Availability Statement

The raw data supporting the conclusions of this article will be made available by the authors, without undue reservation.

## Ethics Statement

Ethical review and approval was not required for the study on human participants in accordance with the local legislation and institutional requirements. The participants provided their written informed consent to participate in this study.

## Author Contributions

RP wrote all the sections of this manuscript.

## Conflict of Interest

The author declares that the research was conducted in the absence of any commercial or financial relationships that could be construed as a potential conflict of interest.

## Publisher’s Note

All claims expressed in this article are solely those of the authors and do not necessarily represent those of their affiliated organizations, or those of the publisher, the editors and the reviewers. Any product that may be evaluated in this article, or claim that may be made by its manufacturer, is not guaranteed or endorsed by the publisher.

## Appendix

**TABLE A1:** Assessment of normality.

Variable	Min	Max	Skew	C.R.	Kurtosis (β 2)	C.R.
Sex	1	2	0.22	1.54	–1.95	–7.01
RL1	1	5	–0.71	–5.06	0.20	0.70
RL2	1	5	–0.66	–4.75	–0.10	–0.37
RL3	1	5	–0.56	–4.01	–0.11	–0.39
RL4	1	5	–0.46	–3.31	–0.11	–0.41
RL5	1	5	–0.57	–4.07	–0.16	–0.55
CSR1	1	6	0.86	6.19	–0.75	–2.70
CSR2	1	6	0.31	2.25	–1.45	–5.22
CSR3	1	6	0.11	0.76	–1.59	–5.69
CSR4	1	6	1.21	8.65	0.14	0.49
CSR5	1	6	0.93	6.70	–0.52	–1.88
CSR6	1	6	0.67	4.79	–1.00	–3.58
CSR7	1	6	0.64	4.61	–1.03	–3.71
CSR8	1	6	–0.20	–1.41	–1.27	–4.57
CSR9	1	6	–0.82	–5.87	–0.33	–1.17
CSR10	1	6	–0.50	–3.56	–0.84	–3.01
CSR11	1	6	–0.45	–3.23	–1.15	–4.11
CSR12	1	6	–0.30	–2.13	–1.18	–4.25
CSR13	1	6	–0.30	–2.18	–1.10	–3.95
CSR14	1	6	–0.83	–5.99	–0.30	–1.07
CSR15	1	6	–0.79	–5.68	–0.06	–0.20
CSR16	1	6	–1.32	–9.44	1.38	4.94
CSR17	1	6	–1.44	–10.36	1.49	5.34
CSR18	1	6	–0.98	–7.06	0.05	0.18
CSR19	1	6	–1.06	–7.59	0.46	1.63
CSR20	1	6	–1.17	–8.37	0.66	2.38
CSR21	1	6	–0.53	–3.81	–0.82	–2.92
CSR22	1	6	–0.99	–7.10	0.38	1.35
CSR23	1	6	–1.05	–7.50	0.59	2.10
CSR24	1	6	–1.00	–7.20	0.45	1.61
CSR25	1	6	–0.98	–7.03	–0.07	–0.26
CSR26	1	6	–0.64	–4.62	–0.47	–1.69
CSR27	1	6	–1.07	–7.69	0.96	3.44
CSR28	1	6	–1.22	–8.72	1.14	4.08
CSR29	1	6	–1.72	–12.33	3.03	10.88
CSR30	1	6	–1.09	–7.83	0.58	2.09
CSR31	1	6	–1.18	–8.48	0.92	3.31
CSR32	1	6	–1.26	–9.05	1.22	4.38
CSR33	1	6	–1.25	–8.94	1.15	4.12
CSR34	1	6	–1.33	–9.56	1.43	5.13
CSR35	1	6	–1.33	–9.51	1.62	5.80
AOC1	1	7	–1.04	–7.44	0.26	0.93
AOC2	1	7	–0.44	–3.16	–0.97	–3.46
AOC3	1	7	–1.11	–7.96	0.59	2.13
AOC4	1	7	–1.24	–8.87	1.07	3.85
AOC5	1	7	–1.43	–10.27	1.62	5.80
AOC6	1	7	–1.47	–10.53	2.29	8.23
AOC7	1	7	–1.62	–11.64	2.52	9.03
AOC8	1	7	–1.08	–7.78	0.71	2.55
Multivariate					435.74	60.19

## Abbreviations

CSR: corporate social responsibility
RL: responsible leadership
AOC: affective organizational commitment
SIT: social identity theory
SEM: structural equation modeling.
